# Induced Pluripotent Stem Cells for Tissue-Engineered Skeletal Muscles

**DOI:** 10.3390/ijms241411520

**Published:** 2023-07-15

**Authors:** Shudong Zhao, Jishizhan Chen, Lei Wu, Xin Tao, Naheem Yaqub, Jinke Chang

**Affiliations:** 1 Division of Surgery and Interventional Science, University College London, London NW3 2QG, UK; 2 Department of iPS Cell Applications, Kobe University, Kobe 657-8501, Japan

**Keywords:** skeletal muscle, induced pluripotent stem cells, tissue engineering, transplantation therapies, disease modelling, biohybrid muscles

## Abstract

Skeletal muscle, which comprises a significant portion of the body, is responsible for vital functions such as movement, metabolism, and overall health. However, severe injuries often result in volumetric muscle loss (VML) and compromise the regenerative capacity of the muscle. Tissue-engineered muscles offer a potential solution to address lost or damaged muscle tissue, thereby restoring muscle function and improving patients’ quality of life. Induced pluripotent stem cells (iPSCs) have emerged as a valuable cell source for muscle tissue engineering due to their pluripotency and self-renewal capacity, enabling the construction of tissue-engineered artificial skeletal muscles with applications in transplantation, disease modelling, and bio-hybrid robots. Next-generation iPSC-based models have the potential to revolutionize drug discovery by offering personalized muscle cells for testing, reducing reliance on animal models. This review provides a comprehensive overview of iPSCs in tissue-engineered artificial skeletal muscles, highlighting the advancements, applications, advantages, and challenges for clinical translation. We also discussed overcoming limitations and considerations in differentiation protocols, characterization methods, large-scale production, and translational regulations. By tackling these challenges, iPSCs can unlock transformative advancements in muscle tissue engineering and therapeutic interventions for the future.

## 1. Introduction

Skeletal muscle, which comprises approximately 40% of total human body weight, is a highly specialized tissue responsible for generating force and participating in human locomotion, posture maintenance, and respiration control [1]. Skeletal muscle plays a crucial role in glucose homeostasis and protein metabolism [2,3]. Following minor injuries like lacerations and sprains, skeletal muscle exhibits remarkable regenerative ability. This process relies on the activation of skeletal muscle stem cells (satellite cells) which undergo multiple rounds of cell division and differentiation into myoblasts. These myoblasts subsequently fuse with existing muscle fibers or with each other to repair damaged muscle tissue. However, severe injuries resulting in volumetric muscle loss (VML) often lead to stem cell loss, compromising the regenerative capacity of the muscle.

**Figure 1:**
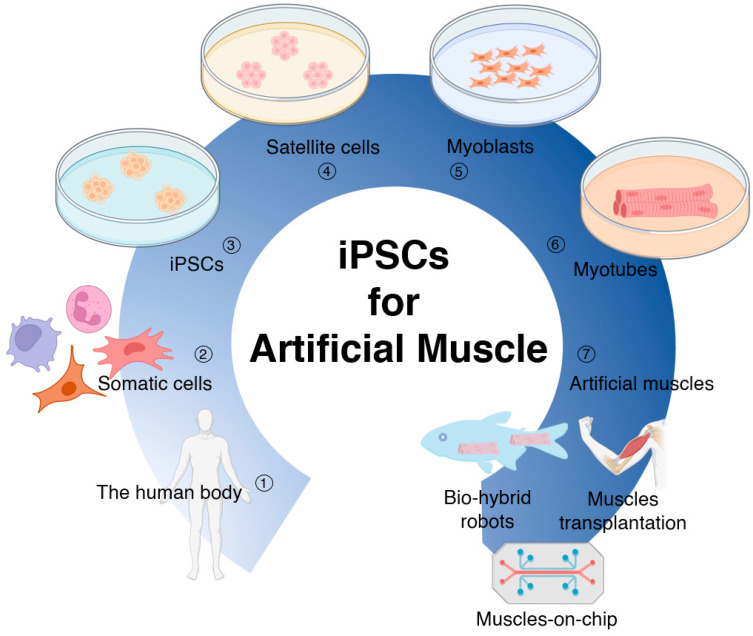
Technical pathways of tissue-engineered iPSCs for artificial muscle applications.

The field of tissue-engineered artificial muscles holds great promise for addressing lost or damaged muscle tissue. These engineered muscles can function as replacements or augmentations, thereby restoring muscle function and enhancing patients’ quality of life. However, a key challenge in developing engineered muscle tissue lies in the availability and sourcing of appropriate cells. Satellite cells are commonly used as the primary cell source for muscle tissue engineering, but their acquisition can be challenging due to limited donor availability and the complex processes involved in isolation, purification, and in vitro expansion, despite the existence of some effective protocols. An alternative approach that has garnered significant attention is the utilization of induced pluripotent stem cells (iPSCs) as a readily available and abundant cell source. iPSCs can be cultured under optimal conditions to generate muscle satellite cells and induce myogenic differentiation, enabling the construction of tissue-engineered artificial muscles with potential applications in transplantation, disease modelling for drug screening, and bio-hybrid robots (Figure 1).

iPSCs have emerged as a promising tool in regenerative medicine and tissue engineering since their discovery in 2006 by Shinya Yamanaka and his colleagues [4]. They provide a new source for tissue engineering- and cell-based therapies without the ethical concerns associated with the use of embryos. iPSCs possess crucial characteristics, including pluripotency, allowing them to differentiate into various cell types found in the body, such as neurons [5], cardiovascular and hematopoietic lineages [6], cardio myocytes [7], and myogenic precursors [8]. These pluripotent properties make iPSCs an appealing candidate for generating tissue-engineered muscles with applications in transplantation, disease modelling for drug screening, and bio-hybrid robots. iPSCs also exhibit self-renewal capacity, enabling symmetrical division and the production of a large number of cells while maintaining their pluripotent properties. Furthermore, iPSCs can be derived from various tissue sources, including skin fibroblasts [9,10], peripheral blood mononuclear cells [11,12], and urinary cells [13,14] (Figure 1). This variety of tissue sources offers a potential solution to the shortage of donor organs and tissues. Patient-specific iPSCs can be utilized to generate satellite cells (myogenic progenitor cells, MPC) and artificial muscle tissues that are not susceptible to immune rejection, overcoming a significant hurdle in traditional allogeneic transplantation methods and eliminating the need for immunosuppressive drugs, thereby improving patients’ postsurgical quality of life.

The use of iPSC-based artificial muscle offers significant advantages in the field of drug discovery, particularly in the areas of personalized medicine and animal welfare. Conventional drug discovery methods heavily depend on animal models, which often fall short in accurately reproducing human responses and raise ethical concerns. In contrast, iPSCs provide a promising alternative by enabling the generation of patient-specific muscle cells that closely mimic human physiology. These iPSC-derived muscle models serve as invaluable tools for assessing drug effectiveness and toxicity, facilitating the development of personalized medicine approaches that account for individual variations in drug response. By harnessing iPSC-derived artificial muscles, the reliance on extensive animal testing can be mitigated, thereby promoting improved animal welfare and a more ethical approach to drug development. Furthermore, the utilization of iPSC-based models permits the investigation of rare diseases and patient-specific genetic variations, offering critical insights into disease mechanisms and supporting the advancement of targeted therapies. The integration of iPSC technology and tissue engineering holds tremendous potential in advancing drug discovery practices and ushering in a paradigm shift in personalized medicine.

Additionally, the development of bio-hybrid artificial muscles based on advanced materials and iPSC-derived muscles represents an emerging direction in iPSC application for muscle tissue engineering. These bio-hybrid systems aim to combine biological and synthetic materials to create more robust and functional artificial muscles. By incorporating iPSC-derived muscle cells into these systems, it is possible to create complex and functional muscle constructs with potential applications in soft robotics and bio-hybrid prostheses.

While the use of iPSCs in tissue-engineered muscles offers many potential advantages, there are also several challenges that need to be addressed. One major hurdle is the development of safe and efficient methods for iPSC generation. Current methods utilizing viruses for iPSC generation may introduce unwanted genetic mutations or tumorigenicity. Hence, alternative non-viral or chemical-based reprogramming strategies are being explored. The undifferentiated state of iPSCs also presents a risk of teratoma formation, which involves the development of tumors containing various cell types. Although current differentiation protocols yield relatively pure populations of specific cell types, residual undifferentiated iPSCs may still pose a risk of tumor formation [15,16,17]. Moreover, translating iPSC technology into clinical settings poses challenges such as high costs and time requirements for iPSC generation and differentiation. The standardization of iPSC generation and differentiation protocols is also necessary to ensure the consistent quality and safety of the final product. Although regulations can present challenges and delays, including extensive preclinical testing, regulatory body approval, and ethics committee clearance, they are essential for guaranteeing the safety and efficacy of new therapies before their application in patients.

This review aims to provide a comprehensive overview of the current state of research on the use of iPSCs in tissue-engineered artificial muscles. We will explore the latest advancements in iPSC generation and differentiation methods, their applications in generating functional muscle structures, drug discovery, and bio-hybrid muscles based on iPSCs. Furthermore, we will discuss the advantages and challenges associated with translating this technology into robotic, medical, and clinical applications.

## 2. Generating iPSCs from Enriched Cell Sources

**Figure 2:**
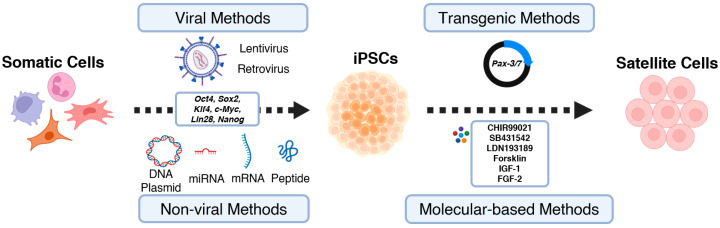
Methods and pathways of generating iPSCs and further differentiation to satellite cells.

The initial reversion of somatic cells to iPSCs was accomplished by Shinya Yamanaka through the transfer of four transcription factors. These factors, collectively known as the Yamanaka factors, included *Oct4*, *Sox2*, *Klf4*, and *c-Myc* [4]. Over time, additional transcription factors have been explored. For instance, the successful reprogramming of human embryonic and fibroblast cells into iPSCs has been achieved by introducing a combination of factors, including *Oct4*, *Sox2*, *Lin28*, and *Nanog* [18]. *Oct4* and *Sox2* are particularly critical for the early development of embryonic stem cells [19] and play key roles in maintaining pluripotency [20,21]. The expression of these transcription factors initiates the reprogramming process by transitioning somatic cells from a differentiated state to a pluripotent state (Figure 2).

Various strategies have been employed for delivering transcription factors in somatic cell reprogramming and iPSC generation, including retroviral [4,9,22], lentiviral [10,23,24,25,26,27], and non-viral methods [18,28,29,30,31,32,33,34,35,36,37,38] (Figure 2). Retroviral and lentiviral approaches utilize viral vectors to introduce reprogramming factors with high efficiency, reaching up to 1% transduction efficacy [9,10,22,23,25,26,27]. However, viral vector-based methods have drawbacks, such as non-specific integration into the genome, potentially leading to genetic mutations and compromised iPSC quality and safety [4,9]. Concerns about immunogenicity, tumorigenicity, and immune responses also arise [39,40,41,42]. 

To overcome viral vector limitations, non-viral methods based on biological molecules such as recombinant plasmid DNA [28,29], mRNA [38], miRNA [36,37], protein [33], or chemical molecules including valproic acid, BIX-01294, and BayK8644 [30,31,32,35] have been developed. While non-viral methods generally exhibit lower efficiency [28,29,34], they offer enhanced safety by reducing the risk of genetic mutations and chromosomal abnormalities [29,33]. Non-viral methods also hold potential for generating clinical-grade iPSCs that meet regulatory requirements.

Both the efficiency and quality of iPSC generation are influenced by several factors. The somatic cell source plays a crucial role, with endothelial cells showing higher reprogramming efficiency than dermal fibroblasts [43]. Donor cell age impacts reprogramming outcomes, with increased risks of exonic mutations and abnormalities in iPSCs with older donors [44]. Culture conditions significantly affect efficiency and quality, requiring optimal nutrient supply and signaling cues for cell survival, proliferation, and pluripotency. Feeder cells, growth factors, and extracellular matrix components are often used. Three-dimensional culture systems and bioreactors enhance iPSC differentiation potential and tissue engineering suitability.

## 3. From iPSCs to Tissue Engineered Artificial Muscles 

Considering the crucial role of MPC in muscle regeneration, the efficient differentiation of iPSCs into MPC is essential for building tissue-engineered artificial muscles. Various protocols and techniques have been employed to induce the generation of iPSC-derived MPC (iPSC-MPC). One commonly used approach is the gene-based approach, where key genes involved in satellite cell development and myogenic differentiation are introduced or modified. For instance, *Pax-7*, functioning in satellite cell specification and function [45], has been delivered into iPSCs to activate the myogenic program, leading to MPC generation [46,47,48] (Figure 2). Similarly, *Pax-3*, a homolog of *Pax-7*, also plays a role to confer myogenic fate in embryogenesis [49]; thus, combining transfection of *Pax-7* and *Pax-3* were also applied to manipulate the iPSC differentiation to MPCs [50] (Figure 2). Although this approach provides a powerful means to direct the fate of iPSCs toward the skeletal muscle lineage, the influence of inserted external genes on cell function and potential mutations needs to be considered. The success of transgenic approaches relies on the efficient monitoring and selection of cells expressing the desired myogenic markers during differentiation.

In contrast to the gene-based approach, non-transgenic methods have been developed to modulate iPSC differentiation into Pax-7^+^ MPC through systematic exposure to specific molecules. These molecules target the critical signaling pathways involved in myogenesis (Figure 2). Typically, iPSCs are first proliferated to an appropriate confluence in a basal medium, followed by a switch to differentiation media supplemented with chemical molecules such as CHIR99021 [8,51,52,53,54,55,56,57] and LDN193189 [8,51,57]. These molecules separately inhibit the glycogen synthase kinase 3 beta (GSK-3β) activity and bone morphogenetic protein (BMP) signaling, collectively activating myogenic signaling cascades and inducing iPSC differentiation into MPC [58,59,60]. During the differentiation process, culture conditions are adjusted at different stages to support cell proliferation and enhance myogenic reprogramming by adding other chemical or biological molecules to the culture media, such as forskolin [61,62], IGF-1 [8,51,52,53,57], or FGF2 [8,51,52,53,54,55,56,57,61,62,63]. Commonly used differentiation protocols are listed in Table 1. These non-gene-based protocols have shown the ability to achieve the differentiation of iPSC-MPC. However, the careful optimization of culture conditions, including medium composition, exposure sequence of molecules, and time duration at each stage, is necessary to ensure the efficient and reliable differentiation of iPSCs into functional skeletal muscle stem cells.

**Figure 3:**
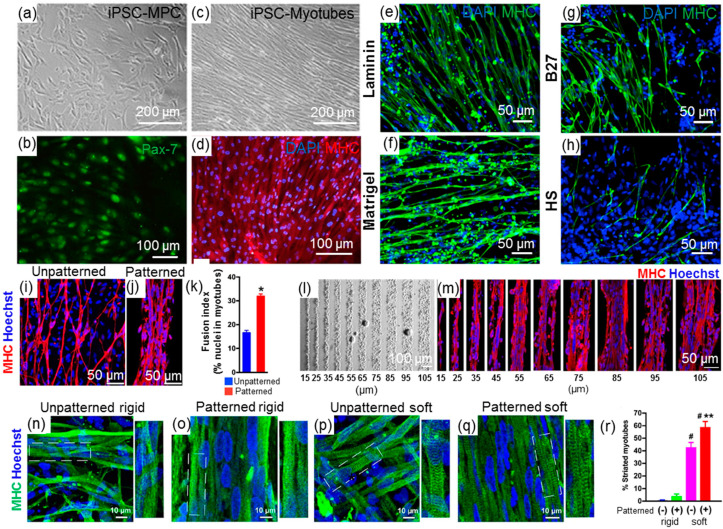
iPSC-derived myotubes and applications in a 2D system. (**a**,**b**) Representative images of iPSC-MPC with the expression of Pax-7. (**c**,**d**) Representative images of iPSC-myotubes with MHC expression, reproduced under the terms of the CC-BY Creative Commons Attribution 4.0 International license [66] Copyright 2021, Springer Nature. (**e**,**f**) Immunofluorescence staining of iPSC-myotubes after 14-day differentiation on the laminin or matrigel coatings. (**g**,**h**) Immunofluorescence staining of iPSC-myotubes at 14-day differentiation in medium with serum-free supplement (B27) or horse serum supplement; images reproduced with permission from the authors of [67] (Copyright 2017, Elsevier). (**i**,**j**) Immunofluorescence staining of iPSC-myotubes at 14-day differentiation on unpatterned or patterned substrates. (**k**) Fusion index evaluation of iPSC-myotubes on unpatterned or patterned substrates, * *p* < 0.01. (**l**,**m**) The iPSC-MPC and iPSC-myotubes linearly aligned on the patterned lanes with a series of widths between 15 and 105 μm. Images reproduced with permission from the authors of [68] (Copyright 2019, Wiley). (**n**–**r**) Physiologically soft surface promotes the formation of myotube striation. (**n**–**q**) Immunofluorescence staining of iPSC-myotubes at 14-day differentiation on soft or rigid substrates. (**r**) Percentage of striated myotubes in various conditions. # *p* < 0.05 compared to unpatterned soft and patterned rigid, and ** *p* < 0.05 compared to unpatterned soft. Images reproduced with permission from the authors of [68] (Copyright 2019, Wiley).

In addition, inducing iPSC-MPC to mature myofibers is also critical in building tissue-engineered artificial muscles. Currently, iPSC-MPC are first expanded onto 2D surfaces coated with extracellular matrix proteins and then induced to differentiate into functional myotubes through starvation methods, typically via culturing in a medium with low horse serum. For example, human iPSCs were manipulated to generate Pax-7^+^ myogenic progenitor cells through sequential treatment with CHIR99021 and givinostat; these cells were then cultured in medium with 2% horse serum for 7 days to differentiate into mature myofibers expressing myosin heavy chain (MHC) (Figure 3a–d) [66]. This approach is relatively simple and easy to implement, enabling clear analysis of the influence of culture parameters on cell behavior, facilitating a better understanding of human muscle development, and providing design cues for artificial muscles. For instance, Jiwlawat et al. studied the influence of different culture surface coating matrices and culture supplements on iPSC-myotube formation. The study demonstrated that laminin coatings exhibited similar effects on myogenic differentiation when compared to matrigel coating (Figure 3e,f) [67]. In addition, the authors found that altering the supplements in the differentiation medium revealed a significant enhancement in myogenesis with the B27 serum-free supplement compared to horse serum (Figure 3g,h) [67]. Moreover, the influence of surface topography and rigidity on myogenesis was also studied in the 2D culture system. iPSC-MPCs were reported to form highly aligned myotubes on patterned surfaces with controllable widths and exhibited a higher fusion index compared to unpatterned platforms (Figure 3i–m) [68]. Furthermore, a higher percentage of striated iPSC-myotubes was observed on physiologically soft surfaces, especially patterned soft surfaces (Figure 3n–r) [68], suggesting the usage of topographical and stiffness cues for designing hybrid muscular tissue in tissue engineering. However, the 2D culture system is unlikely to recapitulate the cellular microenvironment during skeletal muscle development, with limitations in terms of scalability and the ability to create complex muscle structures. Skeletal muscle stem cell niche, a highly designed and complex microenvironment, consists of the extracellular matrix and diverse soluble molecules [69] and plays a critical role in regulating satellite cell growth, maintenance, differentiation, and further muscle regeneration [70,71,72]. Furthermore, biophysical cues of cell niche, including niche stiffness, viscoelasticity, stretching, and interface topography properties, regulate skeletal muscle cells and change cell size and the structural properties of fibers [73,74,75,76,77,78]. All of the above-mentioned niche features highlight the direction for the design of the 3D culture system.

The 3D culture system offers advantages in terms of scalability and the ability to create complex muscle structures. In recent years, the successful differentiation of iPSCs into 3D skeletal muscle organoids has been reported [52,79,80,81,82,83]. For example, Mavrommatis et al. demonstrated the sequential occurrence of multiple myogenic cell types in iPSC-derived organoids, starting from Pax-7^+^ MPCs and progressing to MHC^+^ myotubes [79]. Similarly, Shin et al. generated long-term cultured skeletal muscle organoids and observed the presence of quiescent, non-dividing MPCs during differentiation, suggesting regenerative potential in response to muscle injury [80]. Furthermore, the combination of organoids with functional neuromuscular junctions has led to the development of sensorimotor or neuromuscular organoids, which can stimulate muscle tissue through neural circuits [81,82,83]. Although these 3D organoid models include multiple cell types and allow for the study of the complex muscle interface, they do not fully replicate important architectural features, such as the organized alignment of myofibers. Thus, a scaffold-based 3D culture system was applied to model the architectural features of artificial skeletal muscle. In this approach, iPSCs or iPSC-MPCs are seeded or embedded in pre-designed scaffolds that mimic the extracellular matrix of muscle tissue. Subsequently, they are induced to differentiate into functional myotubes, resulting in the formation of complex artificial muscles. The biomaterials used for scaffold construction can vary from natural materials to artificial materials, such as extracellular matrix proteins and polymers. With the assistance of advanced manufacturing technologies, 3D artificial muscles can be achieved for further applications. The three-dimensional culture of iPSC-derived muscle cells allows for the creation of more complex artificial muscle models that closely resemble the native environment of muscle tissue. These 3D artificial muscle models have been extensively studied for disease modelling, drug discovery, bio-actuator development, and cell therapy.

**Figure 4:**
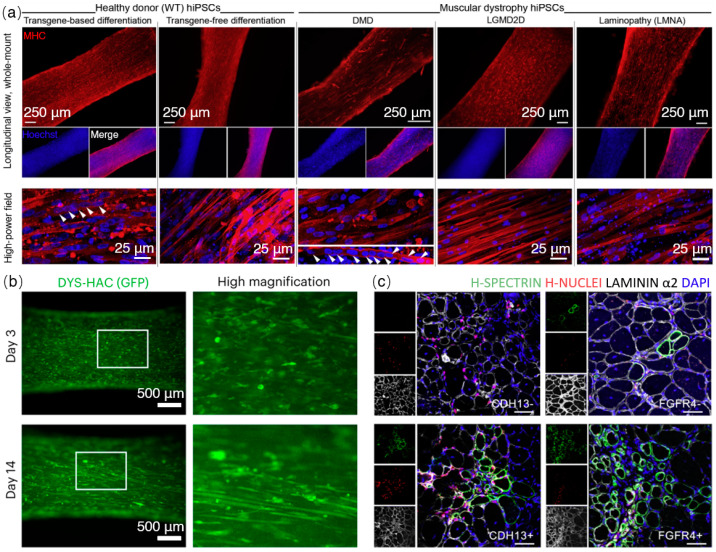
iPSC-derived 3D artificial muscles and applications. (**a**) Whole-mount immunofluorescence staining of MHC on 3D muscle constructs derived from wild-type hiPSCs and dystrophic hiPSCs (DMD, LGMD2D, and skeletal muscle LMNA). Multinucleated myotubes (indicated by white arrows) demonstrate the successful differentiation of iPSCs into functional muscle cells, serving as valuable disease models. Reproduced under the terms of the CC-BY International license [52]. Copyright 2018, Elsevier. (**b**) In vitro real-time monitoring of transgene expression in 3D artificial muscles derived from hiPSCs is achieved using a nonintegrating, nonviral vector. DMD hiPSCs are genetically modified with a human artificial chromosome carrying the complete dystrophin gene locus and a GFP (DYS-HAC) transgene, enabling the assessment of drug effectiveness and the observation of myotube formation during a two-week culture period [102]. Copyright 2023, Springer Nature. (**c**) Assessment of regenerative potential in mdx mice through transplantation of CDH13^+^ and FGFR4^+^ cell populations into cryo-injured tibialis anterior (TA) muscles of NOG-mdx mice. Evaluation of engrafted myofibers for regeneration capacity is conducted four weeks post-transplantation, highlighting the therapeutic potential of these specific cell populations for cell-based therapies, Scale bars: 50 μm. Images reproduced with permission from the authors of [103]. Copyright 2021, Elsevier.

The iPSC-derived muscle tissue can be utilized to model various muscle-related diseases, such as facioscapulohumeral muscular dystrophy [84], myotonic dystrophy [85], limb girdle muscular dystrophy (LGMD) [86,87], infantile Pompe disease [54], amyotrophic lateral sclerosis (ALS) [88,89], Duchenne muscular dystrophy (DMD) [90,91,92,93,94,95,96], and cardiomyopathy [97,98,99], by recapitulating the disease phenotype in vitro. For example, Maffioletti et al. successfully generated 3D skeletal muscle tissue using human iPSCs derived from patients with different types of muscular dystrophies [52]. By inducing myogenic differentiation within tension-induced hydrogels, they achieved aligned myofibers closely resembling human skeletal muscle tissue. Figure 4a illustrates the comparison between healthy and dystrophic muscles. The artificial muscles exhibited the key pathological features of muscular dystrophies and demonstrated successful transplantation into immunodeficient mice. Importantly, the researchers also developed complex, multilineage muscle models by incorporating iPSCs-derived motor neurons, vascular endothelial cells, and pericytes. The study emphasized the potential of the human skeletal muscle organoid-like platform for applications in disease modelling and regenerative medicine.

The application of 3D artificial muscles in drug discovery is also significant. Three-dimensionally constructed artificial muscles based on human iPSCs allow one to test cell toxicity, specificity, and drug efficacies in a humanized isogenic environment [100]. Their application extends beyond disease modelling and encompasses various aspects such as evaluating drugs and therapies, as well as exploring the potential of tissue replacement through in vivo implantation (Table 2). For example, research has shown that human iPSC (hiPSC) derived 3D muscles can be used to monitor gene expression in live tissues using nonviral and viral vectors [101,102] (Figure 4b). Nalbandian et al. conducted researches to optimize the purification of myogenic progenitor cells derived from hiPSCs for transplantation in skeletal muscle diseases [103]. Through the utilization of MYF5 hiPSC reporter lines, the researchers successfully identified CDH13 and FGFR4 as two markers that enable the efficient purification of myogenic cells. The researchers investigated the regeneration capability of cells sorted based on FGFR4 and CDH13 expression in an in vivo setting. Immunohistochemical analysis demonstrated that FGFR4^+^ and CDH13^+^ cells exhibited superior engraftment capacity compared to the negative sorted fractions. Furthermore, FGFR4^+^ cells showed a greater proportion in h-SPECTRIN^+^ (Figure 4c). Transplanted cells purified using these markers demonstrated high regenerative potential and contributed to dystrophin expression restoration in a DMD mouse model. Additionally, the study revealed the regulatory role of MYF5 in CDH13 expression through its binding to promoter regions. These findings underscore the therapeutic potential of purifying hiPSC-MPCs and advancing their applications.

The utilization of iPSC-based 3D artificial muscle models provides substantial advantages in reducing the reliance on animal use for preclinical research, thereby enhancing animal welfare. The patient-specific nature, multicellular composition, and isogenic properties of these models contribute to a decrease in the need for animal testing when validating disease models and evaluating therapies and toxicities. Additionally, iPSC-based 3D artificial muscle represented the most promising strategy for cell therapy, allowing for the replacement of damaged muscle cells in patients. The use of patient-specific iPSCs further enables personalized medicine, as iPSCs derived from an individual’s own cells can be differentiated into muscle cells for transplantation. To construct more complicated tissue structures and improve the functions of iPSC based artificial muscles, biohybrid muscles are pursued based on tissue engineering approaches that represent an emerging direction. 

## 4. Biohybrid Approaches for the Future Artificial Muscles

Biohybrid artificial muscles merge living muscle cells with synthetic materials or supportive structures to better emulate the structure and function of natural muscles, offering substantial advantages in diverse fields such as regenerative medicine, robotics, and biomedical devices. The integration of iPSC-derived muscles further enhances their importance, as iPSCs serve as an ideal cellular source with the ability to be derived from various cell types and enable the creation of patient-specific characteristics. This integration of iPSC-derived muscles opens up new avenues for personalized medicine, disease modelling, and the development of innovative therapies, propelling the field of biohybrid artificial muscles towards remarkable advancements and applications.

**Figure 5:**
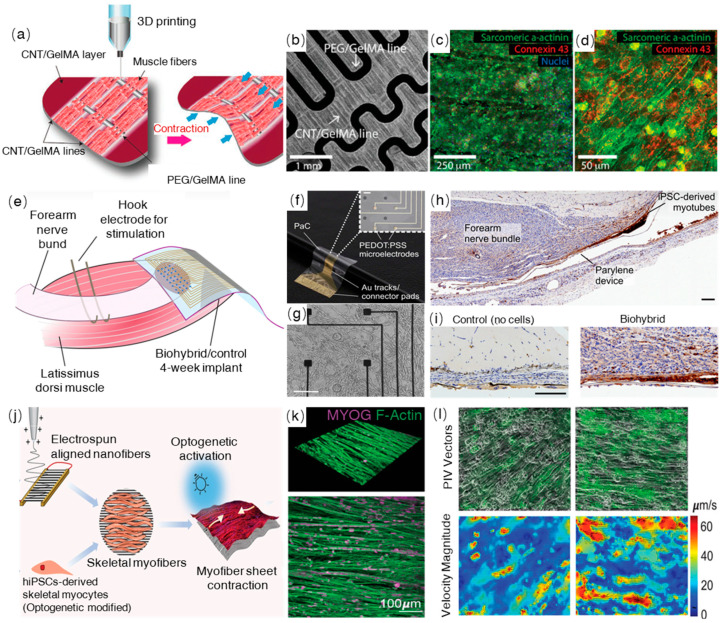
Biohybrid muscles and their applications. (**a**) Design of 3D-printed soft robot with aligned iPSC-derived muscle fibers, enabling upward contraction. (**b**) Microscopic image of the biohybrid robot. (**c**,**d**) Confocal images from the immunostained iPSC-CMs on the soft robot on day 6. Images reproduced with permission from the authors of [112]. Copyright 2022, Wiley. (**e**) Schematic representation of iPSC-derived myocytes as biological targets for peripheral nerve inputs in the novel neural interface. (**f**) A bright field image of the biohybrid electrodes; scale bar: 60 μm. (**g**) Bright-field microscopy image of human iPSC-derived myocytes at day 8 of culture, demonstrating their morphology; scale bars: 465 μm. (**h**) Immunohistochemical staining of human nucleoli (red/brown) in the biohybrid device after 28 days of implantation; scale bar: 50 μm. (**i**) Close-up images comparing control implants (lacking iPSC cells) to biohybrid implants 28 days post-implantation; scale bar: 50 μm. Reproduced under the terms of the CC-BY Creative Commons Attribution 4.0 International license [113]. Copyright 2023, Springer Science. (**j**) Schematic representation of electrospun bio-elastomer nanofibers guiding the differentiation of optogenetically modified iPSC-derived skeletal myofibers. (**k**) Three-dimensional and two-dimensional immunocytochemistry images of skeletal myofibers on aligned nanofibers on day 14 cultures; scale bar: 100 μm. (**l**) Particle image velocimetry (PIV) analysis depicting vector plots and a heat map of velocity magnitude during optogenetically controlled contractions at the peak of contraction. Reproduced under the terms of the CC-BY International license [111]. Copyright 2022, Wiley.

The typical applications of these types of artificial muscles are for biohybrid actuators or robots (Table 2). For example, Tetsuka et al., demonstrated the use of iPSCs-derived cardiomyocytes, differentiated on wirelessly powered, stretchable, and lightweight cell stimulators, for the construction of untethered biohybrid soft robots capable of executing remote-controllable swimming motions [112]. The robot achieved a maximum locomotion speed of approximately 580 µm s^−1^, demonstrating robust and enhanced contractibility of the differentiated cardiomyocytes on the cell stimulators, which replicate the native myofiber architecture. This innovative approach holds promise in enabling the advancement of untethered and wireless biohybrid systems, opening doors to various biomedical applications. (Figure 5a–d). Abadi et al. utilized 3D-patterned polydimethylsiloxane substrates to replicate the biophysical and biomechanical characteristics of the native environment, facilitating the differentiation and maturation of iPSC-derived cardiomyocytes (iPSC-CMs) [108]. As a result, the cells demonstrate enhanced maturation, resembling the shape and orientation of mature cells found in the human myocardium. This improvement is attributed to the reorganization of the cytoskeletal network and the regulation of chromatin conformation.

Biohybrid artificial muscles have the potential to serve as interfaces between muscle and neurons, offering a solution to reduce systemic immunosuppression during implantation. For instance, Rochford et al. explored the use of iPSC-derived myocytes as targets for peripheral nerve inputs, creating a neural interface for restoring neurological functions [113]. The long-term survival and functional integration of implanted biohybrid devices, comprising mature myotubes, were observed in freely moving rats. The tissue-electronics interface was enhanced by the addition of an intermediate cell layer, leading to improved resolution and electrical recording in vivo. These findings hold significant implications for regenerative bioelectronics in restorative therapies (Figure 5e–i).

Another emerging application of iPSC-based artificial muscles lies in stimuli-responsive biohybrid actuators. Cheesbrough et al. introduced a novel approach to mimic native skeletal muscle tissue, utilizing iPSC-derived skeletal muscle cells and electrospun aligned nanofiber sheets. This polymer demonstrated hyperelasticity similar to native skeletal muscle, with aligned nanofibers guiding myoblast alignment, promoting sarcomere formation, and enhancing myotube fusion and myofiber maturation. Elastomer nanofibers provided stabilization to optogenetically controlled human-induced pluripotent stem cell-derived skeletal myofibers, resulting in notable enhancements in contraction velocity and specific force compared to conventional culture techniques. This innovative approach provides a valuable skeletal muscle tissue model for disease modelling, drug discovery, and muscle regeneration purposes (Figure 5j–l).

In the realm of biohybrid robotics/actuators, the utilization of iPSC-derived muscles shows immense potential for constructing efficient dynamical systems. In theory, iPSC-derived muscles cells can be an actuator/robotics unlimited cell source for fabricating tissue based actuators compared to primary cells. With the assistant of responsive material systems, the function of the biohybrid robotics could even act better than natural muscles. However, not many researchers demonstrated successful applications in this topic. Examples of Cheesbrough et al.’s work demonstrated valuable trails for biohybrid muscles based on nanofibers and iPSC-derived skeletal muscles that could generate ~1.19 kN/m2W, which is comparable with robotics constructed based on primary muscle cells [111]. Other studies based on iPSC-derived cardiomyocytes have also demonstrated applications in robotics. For examples, Abadi et al. successfully generated mature cardiomyocytes from induced pluripotent stem cells (iPSCs) using a combination of photolithography and cell imprinting techniques with a scaffold that closely mimics the three-dimensional (3D) in vivo environment for enhanced cardiomyocyte differentiation [108]. Chen et al. explored the use of iPSC-derived cardiomyocytes to actuate the M. menelaus wing [109]. Although these cells exhibited weaker actuation force compared to primary cells, their potential value warrants further optimization, highlighting promising prospects in this field. 

The application of iPSC-derived artificial muscles in robotics is significant due to unlimited cell resources and the potential for biohybrids. Recent research in this field is inhibited for a few reasons: (1) The contraction force generated by iPSC-derived skeletal muscles are typically smaller compared to primary skeletal muscles. This can be attributed to factors such as their immaturity, structural and functional differences, and heterogeneity within the cell population. iPSC-derived cells do not fully replicate the natural development and maturation of primary muscle cells, leading to reduced contractile force. Variations in sarcomere organization, contractile proteins, and muscle fiber alignment further contribute to lower force generation. (2) The weak cell/material interface may cause a reduction in the overall actuations. The integration of iPSC-derived skeletal muscle cells with synthetic materials or supportive structures in biohybrid tissues may not fully replicate the native cell-matrix interactions observed in primary muscle tissues. Variations in mechanical properties, surface topography, and biochemical cues between synthetic materials and the natural extracellular matrix can hinder cell adhesion, alignment, and effective contraction, resulting in reduced force generation. (3) The cost of scaling up production for iPSC-derived muscles in large-scale applications presents a significant cost challenge. The existing methods employed for generating iPSC-derived muscles are frequently laborious and time-consuming, which restricts their suitability for mass production. The expenses associated with expanding production to meet the requirements of tissue-engineered robotics can be a major hindrance, particularly when compared to alternative options like primary muscle cells or synthetic muscle-like materials. 

To address these hurdles, the optimization of differentiation protocols, culture conditions, and cell/material interfaces is crucial. Enhancing the integration of iPSC-derived cells with the microenvironment and promoting neuromuscular junction formation can bridge the gap in contraction force. Biomimetic materials, improved electrical conductivity, and functional interfaces are key to achieving enhanced force generation in biohybrid iPSC-derived skeletal muscle tissues. Moreover, streamlining production processes and reducing costs are both essential for the feasibility of large-scale iPSC-derived muscle applications in tissue-engineered robotics. These strategies will drive progress in tissue engineering and make iPSC-derived muscles more practical and cost-effective for wider implementation. 

## 5. Discussion

The selection of an iPSC generation method for artificial muscles depends on various factors, such as the cell type being reprogrammed, the method’s efficiency, and its potential impact on iPSC quality and safety. The development of safe and efficient methods for iPSC generation is crucial for translating iPSC technology into applications for artificial muscles. 

Similarly, the selection of a iPSC-based artificial muscle generation method is critical in achieving the desired functionality and resemblance to native muscle tissue. Differentiation protocols have been developed to guide iPSCs toward a muscle lineage, including the use of transgenic methods and molecular-based approaches. These methods aim to recapitulate the developmental processes involved in muscle formation and maturation. However, there is still a need for the development of novel differentiation protocols that can produce functional muscle cells with high efficiency and consistency.

The utilization of iPSCs offers several advantages for the future of tissue-engineered artificial muscles. Two-dimensionally cultured iPSC-myotubes provide a simple and easily implementable system that facilitates the analysis of culture parameters on cell behaviors. In contrast, iPSC-based skeletal muscle organoids offer a 3D structure with multiple cell types, enabling the study of interactions between muscle and adjacent tissues. The integration of iPSC-derived muscles with biohybrid approaches, such as incorporating functional synthetic materials or responsive structures, better emulates the features and functions of skeletal muscles in vivo. One significant advantage is the generation of iPSCs from patient-specific cells, which facilitates the development of personalized drug discovery and patient-specific skeletal muscle alternatives. These advancements hold great promise for applications in regenerative medicine, soft robotics, and biomedical devices.

**Figure 6:**
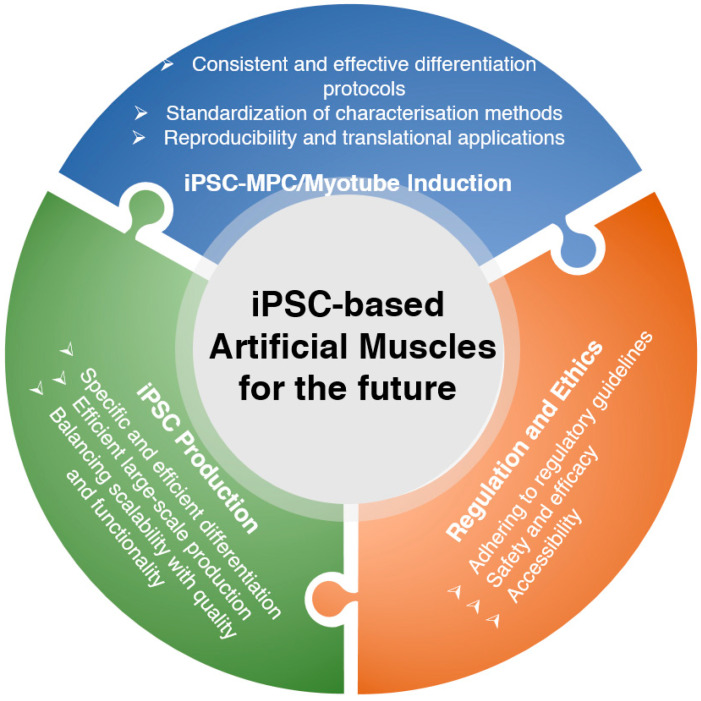
Current hurdles of and future directions for iPSC-derived artificial muscles.

However, there are certain limitations that need to be addressed in the field of iPSC-based tissue-engineered artificial muscles in the future (Figure 6). Firstly, the development of novel differentiation protocols is still ongoing, and further optimization is needed to improve the efficiency and consistency of muscle cell differentiation from iPSCs. Standardized protocols that can generate functional muscle cells with high reproducibility are essential for widespread adoption and clinical translation. In addition, improved characterization methods are required to accurately assess the maturity and functionality of iPSC-derived muscle cells. Currently, there is a lack of reliable markers and assays that can comprehensively evaluate the structural and functional properties of these cells. Efforts should be made to establish standardized protocols for characterizing iPSC-derived muscles, allowing for better comparisons and evaluations of different studies. 

Another challenging research direction is the specific and efficient differentiation of iPSCs into desired muscle cell types. The current differentiation protocols often result in heterogeneous cell populations with varying degrees of muscle cell characteristics. Efforts should be directed towards improving the specificity and efficiency of differentiation, aiming for the generation of highly pure populations of functional muscle cells. Furthermore, efficient large-scale production methods for iPSC-derived muscle cells need to be developed. The current methods often involve labor-intensive and time-consuming processes, which are not scalable for industrial production. Efficiently streamlining and automating the production process while ensuring the quality and functionality of the cells is imperative for the practical implementation of iPSC-based artificial muscles.

As iPSC technology continues to progress, the possibility of commercializing iPSC-based therapies and treatments becomes apparent. Ethical concerns arise surrounding the accessibility and affordability of these treatments, emphasizing the importance of ensuring they are accessible to a diverse patient population rather than being limited to those with financial means. Striking a balance between commercial interests and equitable access to iPSC-based therapies becomes a crucial ethical consideration in order to promote fairness and inclusivity. Regulatory limitations also need to be considered in the development of iPSC-based tissue-engineered artificial muscles. The isolation and sterilization of iPSCs and their derivatives should adhere to strict regulatory guidelines to ensure safety and quality. Compliance with regulatory requirements is necessary for the successful translation of iPSC-based technologies from the laboratory to clinical settings.

## 6. Conclusions

In conclusion, the selection of iPSC generation and iPSC-based artificial muscle generation methods are critical for the successful development of tissue-engineered artificial muscles. The advantages of using iPSCs, including their preferable cell sources, potential for patient-specific drug discovery, and future improvements in human muscle function and neuromuscular junctions, hold great promise for the field. However, several limitations need to be addressed, including the development of novel differentiation protocols, improved characterization methods, and efficient large-scale production methods. Regulatory considerations and functional limitations, such as unspecific differentiation, should also be taken into account for the practical application and clinical translation of iPSC-based artificial muscles.

## Figures and Tables

**Table 1:** Molecular-based approaches for the differentiation of iPSC-MPC.

Ref.	Myogenic Progenitor Cell Differentiation Process		Markers
Hosoyama et al., 2014 [64]	Stemline medium, FGF2 (100 ng/mL), EGF (100 ng/mL), heparin sulfate (5 ng/mL)	42 days	GE: *PAX-3*, *PAX-7*
			PE: PAX-7
Chal et al., 2016 [57]	DMEM/F12 medium, penicillin/streptomycin (2%), NEAA (1%), ITS (1%), CHIR99021 (3 μM), LDN193189 (0.5 μM), FGF2 (20 ng/mL) (FGF2 added at day 4);	6 days	PE: PAX-7, MYOG
	DMEM/F12 medium, penicillin/streptomycin (2%), NEAA (1%), knockout serum replacement (15%), 2-ME (0.1 mM), IGF-1 (2 ng/mL), HGF (10 ng/mL), FGF2 (20 ng/mL), LDN193189 (0.5 μM);	2 days	
	DMEM/F12 medium, penicillin/streptomycin (2%), NEAA (1%), knockout serum replacement (15%), 2-ME (0.1 mM), IGF-1 (2 ng/mL), HGF (10 ng/mL) (HGF added at day 5);	22 days	
Iovino et al., 2016 [62]	STEM Diff Apel medium, FGF2 (10 ng/mL), BIO (0.5 μM), forskolin (20 mM) (FGF2, BIO, and forskolin added at day 1, 3, and 5);	7 days	GE: *PAX-7*, *MYF5*, *MYOD1*
Shelton et al., 2016 [55]	E6 medium, CHIR99021 (10 µM);	2 days	GE: *MYF5*, *MYOD1*, *MYOG*
	E6 medium;	10 days	
	StemPro-34 medium, 1-thioglycerol (0.45 mM), gentamicin (5 µg/mL), L-glutamine (2 mM), transferrin (10.7 µg/mL), FGF2 (5 ng/mL);	8 days	
	E6 medium;	15 days	PE: PAX-7, MHC2
	DMEM/F12 medium, ITS (1%), N-2 Supplement (1%), gentamicin (0.01%)	15 days	
Swartz et al., 2016 [56]	IMDM/F12 medium, bovine serum albumin (5 mg/mL), lipids (1X), transferrin (15 µg/mL), 1-thioglycerol (450 µM), insulin (7 µg/mL), FGF2 (20 ng/mL), LY294002 (10 µM), BMP4 (10 ng/mL), CHIR99021 (10 µM) (BMP4 and CHIR99021 were removed after 1.5 days)	7 days	GE: *PAX-3*, *MYOG*, *MYOD1*
	MB-1 medium, fetal bovine serum (15%);	5 days	PE: PAX-7, MYOG
	DMEM medium, horse serum (2%);	10 days	
	DMEM/F12 medium, N-2 supplement (1%), ITS (1%)	7–10 days	
Sun et al., 2022 [65]	N2 medium, CHIR99021 (3 µM);	4 days	PE: PAX-7
	N2 medium, DAPT (10 µM).	8 days	

2-ME, 2-mercaptoethanol; BIO, 6-bromoindirubin-3′-oxime; DAPT, an inhibitor of γ-secretase; DMEM, Dulbecco’s Modified Eagle Medium; EGF, Epidermal growth factor; FGF2, Fibroblast growth factor 2; GE, Gene expression; HGF, Hepatocyte growth factor; IGF-1, Insulin-like growth factor-1; IMDM, Iscove’s Modified Dulbecco’s Medium; ITS, Insulin-transferrin-selenium; LY294002, Phosphoinositide 3-kinase inhibitor; MYF5, Myogenic factor 5; MHC, Myosin heavy chain; MYOD1, Myoblast determination protein 1; MYOG, Myogenin; NEAA, Non-essential amino acid; PAX, Paired box; PE, Protein expression.

**Table 2:** iPSC-derived artificial skeletal muscle for different applications.

Applications	Cell Type	Culture Environment	Type of Study	Ref.
Disease model	Human iPSCs, induced PAX-7	3D Myobundles anchored within nylon frame	in vitro/in vivo	Rao et al., 2018 [47]
			immunodeficient mice model	
Disease model	Human iPSC-derived skeletal myoblasts (+/− MN Coculture)	Collagen/Matrigel-myoblastmix, pillar anchored, microfluid device	in vitro model	Osaki et al., 2018 [104]
Disease model	Human iPSC line NCRM1	Matrigel	in vitro model	Al Tanoury et al., 2021 [105]
Disease model	Human iPSC derived from dermal fibroblasts (Facioscapulohumeral muscular dystrophy patient)	Matrigel	in vitro model	Sasaki-Honda et al., 2018 [106]
Disease model	Human iPSC derived from miyoshi myopathy patient	Collagen I/Matrigel	in vitro model	Tanaka et al., 2013 [107]
Drug screening	Human iPSC MyoD transfection	Matrigel	in vitro model	Uchimura et al., 2017 [96]
Actuator/robotics	Human iPSC	PDMS micro pattern	in vitro model	Abadi et al., 2018 [108]
Actuator/robotics	Rat iPSC	Natural micropattern	in vitro model	Chen et al., 2019 [109]
Actuator/robotics	Human iPSCs induced Myoblasts	PDMS micro-posts	in vitro model	Yoshioka et al., 2021 [110]
Actuator/Biohybrid Robotics	Human iPSC, induced PAX-7	Suspended elastomer nanofibers	in vitro model	Cheesbrough et al., 2022 [111]

## Data Availability

No new data were created during this study.
